# Isolation and characterization of multiple drug resistance bacterial pathogens from waste water in hospital and non-hospital environments, Northwest Ethiopia

**DOI:** 10.1186/1756-0500-7-215

**Published:** 2014-04-05

**Authors:** Feleke Moges, Mengistu Endris, Yeshambel Belyhun, Walelegn Worku

**Affiliations:** 1 Department of Medical Microbiology, College of Medicine and Health Sciences, University of Gondar, Gondar, Ethiopia; 2 Department of Immunology and Molecular Biology, College of Medicine and Health Sciences, University of Gondar, Gondar, Ethiopia; 3 Institute of Virology, Faculty of Medicine, University of Leipzig, Leipzig, Germany; 4 Department of Environmental and Occupational Health and Safety, College of Medicine and Health Sciences, University of Gondar, Gondar, Ethiopia

**Keywords:** Hospital environment, Multiple drug resistance, Non-hospital environment, Waste water

## Abstract

**Background:**

The importance of bacterial isolates from waste water environment as a reservoir of antibiotic resistance and a potential source of novel resistance genes to clinical pathogens is underestimated. This study is aimed at to isolate and characterize public health important bacteria from waste water in hospital and non- hospital environments and evaluate the distribution of multiple drug resistance bacteria in the study area.

**Methods:**

A cross-sectional study was conducted at Gondar from January-June 2012. The hospital waste water was taken from different sections of the Gondar University Teaching Hospital. Non- hospital environment samples were taken at different sites of the university campuses, Gondar College of Teachers education, and soft drink factory in Gondar. Samples were aseptically collected, transported and processed with in two hours following standard procedure. Identified organisms were assessed for different antibiotics following Kirby-Bauer disk diffusion method. All data was registered and entered in to SPSS version 16 computer program. P-values less than 0.05 were taken as statistically significant.

**Result:**

A total of 60 waste water samples were processed for the presence of drug resistance pathogens. Among the total samples 113 bacterial isolates were recovered and of these 65 (57.5%) were from hospital environment and 48 (42.5%) were from non-hospital environment. The most frequently identified bacterium was *Klebsiella* spp. 30 (26.6%) followed by *Pseudomonas* spp. 19(16.8%), *E. coli* (11.5%) and *Citrobacter* spp (11.5%), and *Staphylococcus aureus* (8.2%). The over all prevalence of multiple drug resistance (MDR) in this study was 79/113 (69.9%). MDR in hospital environment was found to be 53/68 (81.5%) while in non hospital environment was found to be 26/48 (54.2%).

**Conclusions:**

Multiple drug resistance to the commonly used antibiotics is high in the study area. The contamination of waste water by antibiotics or other pollutants lead to the rise of resistance due to selection pressure. The presence of antibiotic resistance organisms in this waste water should not be overlooked. Since this organisms may be vital to the safety and well-being of patients who are hospitalized and individual susceptible to infection. Therefore, proper waste water treatment plant should be established and improved sanitary measure should be practice.

## Background

The use of antibiotics and spread of antibiotic resistance in clinical settings is a well recognized problem, but antibiotics and antibiotic resistance as environmental problems and pollutants have largely been overlooked. As a result, the increasing incidence of resistance to a wide range of antibiotic agents by a variety of organisms is a major concern facing modern medicine.

Hospital wastewater can be hazardous to public health and ecological balance since it can contain many kinds of pollutants such as radioactive, chemical and pharmaceutical wastes and also pathogenic microorganisms [1]. Uncontrolled and excessive use of antibiotics by human and animals results an increase in antibiotic resistance and cause the spread of resistance genes in environmental samples such as hospital waste water [2]. Studies have demonstrated that hospital wastewater is highly selective environments and that they contribute to the high rates of resistant bacteria that are being discharged in the natural environment [3]. As demonstrated by Colomer-Lluch et al., the occurrence of bacteriophages from samples of animals fecal wastes can be environmental vectors for the horizontal transfer of antibiotic resistance genes [4].

Therefore, antibiotic resistance is not only found in pathogenic bacteria but also in environmental organisms inhabiting terrestrial and aquatic habitats. Higher numbers of resistant bacteria occur in polluted habitats compared with unpolluted habitats, indicating that humans have contributed substantially to the increased proportion of resistant bacteria occurring in the environment [5].

Antibiotics exert a selection in favor of resistant bacteria by killing or inhibiting growth of susceptible bacteria; resistant bacteria can adapt to environmental conditions and serve as vectors for the spread of antibiotic resistance [6]. The main risk for public health is that resistance genes are transferred from environmental bacteria to human pathogen [6,7]. The volume of antibiotics used in hospitals and private households and released into effluent and municipal sewage indicates a selection pressure on bacteria [8]. Waste effluent from hospitals contains high numbers of resistant bacteria and antibiotic residues at concentrations able to inhibit the growth of susceptible bacteria [9,10]. As a result, hospital waste effluent could increase the numbers of resistant bacteria in the recipient sewers by both mechanisms of introduction and selection for resistant bacteria [11].

As reported by Gelaw [12] postoperative surgical site infections in the hospital were mainly due to presence of contamination of medical equipment, environmental surfaces, air and hands of health personnel. The knowledge about the risk perception of healthcare workers (HCWs) toward healthcare waste management in health care facilities of Gondar town confirms that, only 60% of the HCWs had adequate risk perception that improperly managed healthcare wastes could transmit infection to HCWs and patients [13]. Practice of self medication was also a problem [14] and injection and antibiotic use of the hospital is not to the standard of WHO limit [15]. It is believed that, these factors may serve as a selective pressure and contribute to the increase drug resistance strains in the hospital environment. In Ethiopia especially, in Northwest Ethiopia there is no data concerning resistance profiles of microorganisms isolated from hospital or community sewage. This study is therefore, attempt to generate original local data and examine the magnitude of drug resistance pathogens in hospital and non-hospital environments in Gondar, Ethiopia.

## Methods

### Study setting and sampling

A cross-sectional study was conducted from January-June 2012 at Gondar University Hospital, Gondar College of Teachers Education, Pepsi soft drink factory and other campuses of the faculty of Social, Natural and Computational Sciences, University of Gondar.

Gondar University Hospital is a tertiary level teaching hospital that provides health service to over five million inhabitants in Northwest Ethiopia, and is located 737 Km North from the capital city, Addis Ababa, Ethiopia.

### Hospital waste water samples

Thirty three untreated wastewater samples were collected from Gondar University hospital at different sites. The superficial wastewater samples were collected at the open surface flowing cross the hospital campus and sample were taken at 100 m upstream of the inlet (site1) and dawn stream that goes out (outlet) the hospital (site 2). The other samples were collected at different outlets within the different section of the hospital and designating as waste water from Intensive Care Unit and Surgery (operation theatre) (site 3); Pathology Laboratory (site 4), Hospital Laboratory and Sterilization room (site 5); Pediatric ward (site 6); Gynecology and Obstetric ward (site 7); Internal Medicine ward (site 8); General ward (site 9); Orthopedic and Surgical ward (site 10).

### Non- hospital waste water samples

Twenty seven waste water samples were collected from four different sources other than sites of the university hospital: Social science faculty (site 11), Natural and computational sciences faculty (site 12), Gondar College of Teacher’s Education (site 13) and Pepsi soft drink factory (Site 14).

### Sample processing, isolation and identification of bacteria

Samples were transported to the laboratory in cool conditions and processed within two hours of collection. A 100 mL aliquot of waste water of serial ten-fold dilutions for each sample was filtered through a 0.22 μm pore membrane (Millipore, Billerica, MA) which was then placed on plate count agar and incubated aerobically at 37°C for 24–48 hours. After incubation, based on colony morphology representative colonies were picked and sub-cultured on different selective and differential media such as blood agar, MacConkey agar, and pseudomonas agar. After obtaining pure colonies and recording important features such as haemolysis on blood agar isolated organisms were identified biochemically in a systematic way following standard methods [16].

### Antimicrobial susceptibility testing

Once the bacteria is isolated and identified from each sample collected, the standard Kirby-Bauer disk diffusion method was used to determine the antimicrobial susceptibility profiles of the isolates [17]. Bacterial inoculum was prepared by suspending the freshly grown bacteria in 4–5 ml sterile nutrient broth and the turbidity was adjusted to that of a 0.5 McFarland standard. The antimicrobial susceptibility testing was performed using Mueller-Hinton medium against ampicillin (10 μg), chloramphenicol (30 μg), ciprofloxacin (5 μg), clindamycin (2 μg), erythromycin (15 μg), gentamicin (10 μg), methicillin (5 μg), vancomycin, (30 μg), streptomycin (10 μg), trimethoprim-sulfamethoxazole (5 μg), tetracycline (30 μg), nalldixic acid (30 μg), cephalothin (30 μg), cefotaxime (30 μg), kanamycin (30 μg). The plates were incubated aerobically at 37°C for 18–24 hours. The zones of inhibition were measured and compared with National Committee for Clinical Laboratory Standards (NCCLS) guidelines [18]. *Escherichia coli* ATCC 25922 and *P. aeruginosa* ATCC 27853 were used for quality control. Data was entered in to SPSS version 16 computer program. P-values less than 0.05 were taken as statistically significant.

## Results

A total of 60 waste water samples were processed for the presence of drug resistance bacterial pathogens. Of these samples 85% of the samples were positive to one or more isolates. Among the total samples 113 bacterial isolates were recovered and 65 (57.5%) were from hospital environment and 48 (42.5%) were from non-hospital environment (Table 1).

**Table 1:** Distribution of samples taken from hospital and non-hospital environments, Gondar 2012

**Sample sites**	**Total samples**	**Sample positive**	**Samples negative**	**Total bacterial isolates recovered**
	**N (%)**	**N (%)**	**N (%)**	**N (%)**
**Hospital environment**	33 (55)	28 (54.9)	5 (55.6)	65 (57.5)
**Non- hospital environment**				
College of teacher’s education	7 (11.7)	7 (13.7)	0	13 (11.5)
Maraki campus	6 (10)	6 (11.8)	0	15 (13.3)
Tewodrose campus	9 (15)	5 (9.8)	4 (44.4)	7 (6.2)
Pepsi soft drink factory	5 (8.3)	5 (9.8)	0	13 (11.5)
Sub total	27 (45)	23 (45.1)	4(44.4)	48 (42.5)
**Overall**	**60 (100)**	**51 (100)**	**9 (100)**	**113 (100)**

Most frequently isolated bacterium was *Klebsiella* spp. 30 (26.5%) followed by *Pseudomonas* spp. 19 (16.8%), *E. coli* (11.5%) and *Citrobacter* spp (11.5%), and *S. aureus* (8.2%). Two *Shigella* species were isolated from hospital environment (Table 2).

**Table 2:** Number of bacteria isolated at each sampling points from hospital and non-hospital environments Gondar, 2012

**Bacterial isolates**	**Hospital env’t**	**Non hospital env’t**	**Total**
	**No. (%)**	**No. (%)**	**No. (%)**
*E. coli*	8 (12.3)	5 (10.4)	13 (11.5)
*Klebsiella* spp*	19 (29.2)	11 (22.9)	30 (26.6)
*Pseudomonas aeruginosa*	14 (21.5)	5 (10.4)	19 (16.8)
*Enterococci* + CoNS**	5 (7.7)	3(6.3)	8 (7.1)
*S. aureus*	5 (7.7)	5 (10.4)	10 (8.8)
*Shigella Spp*	2 (3.1)	-	2 (1.8)
*Citrobacter* spp	5 (7.7)	8(16.7)	13 (11.5)
*Enterobacter* spp	2(3.1)	5 (10.4)	7 (6.2)
Others***	5 (7.7)	6 (12.5)	11 (9.7)
**Total**	**65 (100)**	**48 (100)**	**113 (100)**

*Klebsiella spp. (*K. Pneumoniae* = 23; *K. oxytoca =2; K. ozaenae =5).*

**CoNS = Coagulase Negative *Staphylococci.*

***Others (*Providencia spp = 4; Morganella* spp = 3; *Proteus* spp = 2; *Serratia* sp. =1; *Edwardsiella sp. =1).*

All the isolates of *S. aureus* were resistant to ampicillin (100%). One isolates of *S. aureus* was resistant to 11 antimicrobials including methicillin. However, no isolates of *S. aureus* was recovered to be vancomycin resistant (Table 3). Multiple drug resistance was also common in gram negative isolates to commonly used antibiotics in the study area. All isolates of *E. coli*, *Citrobacter* spp. and *Enterobacter spp.* were 100% resistant to ampicillin. The over all resistance of gram-negative bacteria to ampicillin was 97%, followed by cephalotin 49%, cotrimoxazole 38%, tetracycline 37%, naldixic acid 36%, cefotaxime 33%; least resistant being ciprofloxacin 12% (Table 4).

**Table 3:** Resistance rate of gram positive isolates to commonly used antibiotics in Gondar, 2012

**Bacterial isolates**	**Antibiotic resistance N (%)**														
	**Met**	**Van**	**DA**	**GN**	**SXT**	**ERY**	**TTC**	**CAF**	**CIP**	**NA**	**S**	**AMP**	**CRO**	**KF**	**CTX**
*Enterococci* + CoNS (n = 8)	2 (25)	-	2 (25)	-	2 (25)	3 (38)	2 (25)	2 (25)	1(13)	4 (50)	1(13)	5 (63)	-	-	-
*S. aureus* (n = 10)	1 (10)	-	2 (20)	1 (10)	-	2 (20)	1 (10)	2 (20)	1 (10)	6 (60)	1 (10)	10 (100)	1 (10)	1 (10)	2 (20)
Total (n = 18)	**3 (17)**	**-**	**4 (22)**	**1 (6)**	**2 (11)**	**5 (28)**	**3 (17)**	**4 (22)**	**2 (11)**	**10 (56)**	**2 (11)**	**15 (83)**	**1 (6)**	**1 (6)**	**2 (11)**

MET = Methicillin; VAN = Vancomycin; DA = Clindamycin; GN = Gentamycin; SXT = Cotrimoxazole; ERY = Erytromycin; TTC = Tetracycline; CAF = Chloramphinicol;

CIP = Ciprofloxacin, NA = Naldixic acid; S = Streptomycin; AMP = Ampicillin CRO = Ceftriaxone; KF = Cephalothin; CTX = Cefotaxime.

**Table 4:** Resistance rate of gram negative isolates to commonly used antibiotics in Gondar, 2012

**Bacterial isolates**	**Antibiotic resistance N (%)**											
	**GN**	**SXT**	**TTC**	**CAF**	**CIP**	**NA**	**S**	**AMP**	**CRO**	**KF**	**CTX**	**K**
***E. coli *** **(n = 13)**	1 (8)	5 (38)	5 (38)	1 (8)	3 (23)	4 (31)	1 (8)	13 (100)	2 (15)	3 (23)	3 (23)	2 (15)
***Klebsiella spp *** **(n = 30)**	6 (20)	10 (33)	8 (27)	4 (13)	5 (17)	7 (23)	9 (30)	30 (100)	8 (27)	12 (40)	10 (33)	6 (20)
***Pseudomonas aeruginosa *** **(n = 19)**	1 (5)	11 (58)	10 (53)	7 (37)	1 (5)	10 (53)	3 (16)	18 (95)	3 (16)	16 (84)	11 (58)	6 (32)
***Shigella *** **spp. (n = 2)**	-	-	-	-	-	1 (50)	-	1 (50)	1 (50)	2 (100)	2 (100)	-
***Citrobacter *** **spp (n = 13)**	2 (15)	4 (31)	5 (38)	3 (23)	-	5 (38)	5 (38)	13 (100)	1 (8)	4 (31)	1 (8)	2 (15)
***Enterobacter *** **spp (n = 7)**	1 (14)	3 (43)	4 (57)	1 (14)	1 (14)	2 (29)	2 (29)	7 (100)	1 (14)	3 (43)	1 (14)	1 (14)
**Other G-ve (n = 11)**	2 (18)	3 (27)	3 (27)	1 (9)	1 (9)	5 (46)	3 (27)	10 (91)	3 (27)	7 (64)	3 (27)	3 (27)
**Total (n = 95)**	**13 (14)**	**36 (38)**	**35 (37)**	**17 (18)**	**11 (12)**	**34 (36)**	**23 (24)**	**92 (97)**	**19 (20)**	**47 (49)**	**31 (33)**	**20 (21)**

GN = Gentamycin; SXT = Cotrimoxazole; TTC = Tetracycline; CAF = Chloramphinicol; CIP = Ciprofloxacin, NA = Naldixic acid; S = Streptomycin; AMP = Ampicillin; CRO = Ceftriaxone; KF = Cephalothin; CTX = Cefotaxime; K = Kanamycin.

Among Gram negative bacterial isolates the most striking multiple drug resistant isolate which was resistant to 12 antibiotics tested was *Enterobacter* spp. followed by *Klebsiella* spp. to 11 antibiotics*, Pseudomonas* spp. to 10 antibiotics.

Among 113 isolates 1 (0.9%) was susceptible to all antibiotics tested, 33(29.2%) were resistant only for one antibiotics, 22 (19.5%) were for 2 antibiotics, 9 (7.9%) were for 3 antibiotics, 14 (12.4%) were for 4 antibiotics, 34 (30.1%) resistant to 5 or more antibiotics (Table 5). The over all prevalence of multiple drug resistance (MDR) (two or more drugs) in this study was 79/113 (69.9%). Multiple drug resistance in hospital environment was found to be 53/65 (81.5%) while in non-hospital environment was found to be 26/48 (54.2%). When we consider isolates showing resistant to more than 5 antibiotics, the distribution of MDR isolates in hospital environment still found to be higher than non-hospital environments with the rate of 30/65 (46.2%) and 4/48 (8.3%), respectively.

**Table 5:** Over all rate of multiple drug resistance (resistant to 2 or more antibiotics) in isolates from hospital and non-hospital waste water, 2012

**Study sites**	**Antibiotic resistance N (%)**						
	**R0**	**R1**	R2*	**R3**	**R4**	≥R5**	**Total**
Hospital environment	0	12 (18.5)	9 (13.8)	4(6.1)	10 (15.4)	30 (46.2)	65 (100)
Non-Hospital environment	1 (2.1)	21 (43.8)	13 (27.8)	5 (10.4)	4 (8.3)	4 (8.3)	48 (100)
**Total**	**1 (0.9)**	**33 (29.2)**	**22 (19.5%)**	**9 (7.9)**	**14 (12.4)**	**34 (30.1)**	**113 (100)**

R0 = Susceptible to all antibiotics tested; R1 = Resistance to 1 antibiotic; R2 = Resistance to 2 antibiotics; R3 = Resistance to 3 antibiotics; R4 = Resistance to 4 antibiotics; R5 and above = Resistance to 5 or more antibiotics. *P = value (>2 antibiotics) = (COR 3.74 1.49-9.53, 95% CI, P = 0.001).

**P = value (≥R5 antibiotics) = (COR 9.43, 2.80-35.03; 95% CI, P = 0.00001).

## Discussion

Among the total isolates of bacteria, 65 (57.5%) were from hospital environment and 48 (42.5%) were from non-hospital environments. The rate of isolation of bacterial pathogens in the hospital environment was higher than the non-hospital environment, this was statistically significant (P = 0.01). Similar trends was reported by Guardabassi et al., that factors other than the indiscriminate use of antibiotics in human medicine, animal husbandry, and agriculture may disrupt the microbial balance in favor of resistant bacteria. In particular, wastewater from pharmaceutical plants could play a role in the selection of antibiotic-resistant bacteria in sewage [19].

In the present study frequently identified bacterium was *Klebsiella* spp. 30 (26.6%) followed by *Pseudomonas* spp. 19 (16.8%), *E. coli* (11.5%) and *Citrobacter* spp (11.5%), and *S. aureus* (8.2%). Similar reports by Ekhaise and Omavwoya in Benin hospital showed that the bacterial genera, *Klebsiella, Pseudomonas* and *Serratia* were the most frequently distributed isolates in the hospital wastewater [20]. Similar study in Rio de Janeiro, Brazil reported that the most common multi-resistant extended spectrum beta-lactamase (ESBL) producing isolates from hospital waste water were *Klebsiella pneumoniae*, *Enterobacter cloacae* and *E. coli*[21].

Multiple drug resistance was also common in gram positive isolates to commonly used antibiotics in the study area. All isolates of *S. aureus* were resistant to Ampicillin (100%). One isolates of *S. aureus* was found to be resistant to 11 antimicrobials including methicillin. As indicated by Abulreesh, multidrug resistant staphylococci (*S. aureus* and coagulase negative *Staphylococci*) have been a common problem and recovered from diverse environmental sources, such as drinking water supplies, foodstuffs, the mucosa of humans and farm animals and hospital environments which can be important public health concern [22]. The number of MRSA isolates in this study was lower than reports from municipal waste water in U. S. A [23]*.* This variation may be due to early introduction of methicillin in USA than the place of the present study, Ethiopia.

Multiple drug resistance was also common in gram negative isolates to commonly used antibiotics in the study area. *E. coli*, *Klebsiella* spp. *Citrobacter* spp. and *Enterobacter spp.* were 100% resistant to Ampicillin. This finding is inconsistent from reports in Brazil that the overall, resistance rates were low in the isolates of *E. coli*, *P. aeruginosa*, *K. pneumoniae* and *A. baumannii* and the susceptibility pattern of *E.coli* and *Klebsiella* for ampicillin was found 40% and 70%, respectively [24]. Among all isolates of Gram negative bacteria 97% of the isolates were resistant to ampicillin, followed by cephalotin 49%, cotrimoxazole 38%, tetracycline 37%, naldixic acid 36% cefotaxime 33%.

The resistant pattern of gram-negative isolates for ciprofloxacin was lower in the present study, 12%. This was different from other study done in Bangladesh 100% resistant [25]. The recent circulation of ciprofloxacin in the study area could have contributed to this low prevalence of drug resistance strains. This idea is supported by Moges et al. that, all urinary tract pathogens such as *E. coli*, *Klebsiella* spp. *Citrobacter* spp were 100% susceptible to ciprofloxacin before 10 years in the same study area, where ciprofloxacin start circulating in the market [26]. Multiple drug resistance from 2–12 antibiotics was found to be 79/113 (69.9%). Multi-drug resistance in hospital environment was 53/65 (81.5%) while in non hospital environment was 26/48 (54.2%). Isolates from hospital environment were found 3.74 times resistant for many drugs than isolates from non-hospital environment (COR 3.74 1.49-9.53, 95% CI, P = 0.001). When we consider isolates showing resistant to more than 5 antibiotics, still hospital environment 30/65 (46.2%) show higher resistant pattern than non-hospital environment 4/48 (8.3%). This was statistically significant (COR 9.43, 2.80-35.03; 95% CI, P = 0.00001). Similar finding demonstrate that, waste effluent from hospitals contains high numbers of resistant bacteria and antibiotic residues at concentrations able to inhibit the growth of susceptible bacteria [9,10].

## Conclusions

Multiple drug resistance to the commonly used antibiotics is high in the study area. This was even higher in hospital waste water. The contamination of water by antibiotics or other pollutants lead to the rise of resistance due to selection pressure. The presence of antibiotic resistant organisms in this waste water should not be overlooked. Since this organisms may be vital to the safety and well-being of patients who are hospitalized as well as individuals who are susceptible to infection. Therefore, proper waste water treatment plant should be established and improved sanitary measure should be practice.

## Competing interests

The authors declare that we have no conflict of interest.

## Authors’ contributions

FM designed and prepared the proposal, conduct the field and laboratory work, analyzed data, wrote and revised the manuscript. ME conduct the field and laboratory work, analyzed data, wrote and revised the manuscript. YB Designed the proposal, analyzed data, reviewed the manuscript and edited the language. WW reviewed the manuscript, analyzed data and edited the language. All authors read and approved the final manuscript.
